# Occurrence Patterns of Traumatic Brain Injury Within the Emergency Department and Internal Screening Process Efficacy During the COVID-19 Pandemic: Retrospective Analysis

**DOI:** 10.2196/29513

**Published:** 2022-03-23

**Authors:** Tapasvini Anmol Paralkar, Phoebe Lay, Sawyer Stubbs, Syed Hadi Ahmed, Minha Ghani, Nico Osier

**Affiliations:** 1 College of Natural Sciences The University of Texas at Austin Austin, TX United States; 2 Department of Neurology Dell Medical School Austin, TX United States; 3 McCombs School of Business The University of Texas at Austin Austin, TX United States; 4 College of Liberal Arts The University of Texas at Austin Austin, TX United States; 5 School of Nursing The University of Texas at Austin Austin, TX United States

**Keywords:** COVID-19, coronavirus, pandemic, clinical recruitment, traumatic brain injury, children, participant-focused, recruitment, enrollment, digital screening, brain, EHR, electronic health record, database

## Abstract

**Background:**

Traumatic brain injury (TBI) is one of the leading causes of death in pediatric patients. Continued recruitment of pediatric TBI participants into a biobank amidst the COVID-19 pandemic not only necessitates adaptive changes to traditional recruitment methods but also requires an evaluation of emergency department (ED) utilization by TBI-presenting patients.

**Objective:**

The primary objective of this exploratory retrospective study was to evaluate pediatric TBI-related ED utilization during the pandemic. The secondary objective was to appraise the efficacy of the research team’s internal screening processes.

**Methods:**

Potential participants (ie, individuals who met all inclusion criteria and would be approached by a consenter) were screened from an ED’s electronic health record system. Data regarding their visit were recorded in a Health Insurance Portability and Accountability Act–compliant manner, which were cleaned through Google Sheets. Cleaned data were then coded as either a screening variable or a hospital utilization variable to examine the effects of the pandemic on internal operations and hospital utilization patterns. The variables were compared between select months during the pandemic in 2020 to analogous months in 2019 in the R programming language via the two-sample Student *t* test and the Mann-Whitney-Wilcoxon rank-sum test.

**Results:**

The sample (N=2321) consisted of 1245 entries from 2019 and 1076 entries from 2020. A significantly greater proportion of potential participants (*P*<.001) were identified in 2020 (222/633, 35.1%) than in 2019 (195/908, 21.4%). A significantly greater proportion of potential participants (*P*<.001) had a visit reason indicative of a TBI in 2020 (181/222, 81.5%) than in 2019 (103/195, 52.8%). A significantly greater proportion of these injuries (*P*=.02) occurred inside (39/181, 21.5%) in 2020 than in 2019 (11/103, 10.7%). No significant difference was found across the mechanism of injury categories reported for potential participants between 2019 and 2020. Potential participants were significantly older (*P*=.006) in 2019 (mean 8.93 years) than in 2020 (mean 7.31 years). Screeners spent significantly longer (*P*=.03) to identify potential participants in March 2020 (55 minutes) than in March 2019 (32 minutes), but spent significantly less time (*P*=.01) to do so in July 2020 (22 minutes) than in July 2019 (42 minutes). Screening coverage was significantly lower (*P*<.001) in March 2020 (241.8 hours) than in March 2019 (346.5 hours). Screening coverage was significantly greater (*P*<.001) in April 2020 (611.5 hours) and July 2020 (513.5 hours) than in April 2019 (470.5 hours) and July 2019 (404.3 hours), respectively.

**Conclusions:**

There was a significant increase in the rate of incoming TBI cases to the ED during the COVID-19 pandemic, warranting continued enrollment with added safety measures. Additionally, refinement of internal processes improved the accuracy of data collection. As demonstrated in this study, researchers can leverage ongoing data collection to facilitate process improvements and evaluate the impact of unexpected global events on their research.

## Introduction

Traumatic brain injury (TBI) is defined as an alteration in brain function or pathology caused by an external force [1]. TBIs most frequently result from contact sport injuries, falls, and motor vehicle accidents (MVAs) [2]. The clinical diagnosis of TBI is made through observation of symptoms, including neurological deficits, loss of consciousness, memory loss surrounding the event, or alteration in mental state at the time of injury [2]. TBIs are more common in the age groups of 0-4 years, 15-19 years, and ≥75 years, indicating that a large majority of TBI occurrences are found in the pediatric population; notably, TBI is the leading cause of death or other negative outcomes in the pediatric population [3].

Over 812,000 emergency department (ED) visits for pediatric TBI occurred in 2014, suggesting that many parents or guardians may bring their child to the ED first, instead of making an appointment with their primary health care provider [4]. Additionally, significant increases in ED visits for pediatric TBI suggest that there are more focused efforts in referring children with suspected TBI primarily to the ED [5]. However, there remains an important gap regarding how to maximize recruitment of ED patients into pediatric TBI biobanks. This knowledge gap has only widened with the development of COVID-19. The parent study, which focuses on enrollment of pediatric TBI patients into biobanks, initially halted recruitment because in-person enrollment was critical for proper consent and biospecimen procurement; however, the research team has maintained existing digital screening efforts to identify potential participants, as described in the Methods section below. It is critical to continue TBI research in pediatric populations while additionally examining the effects of the pandemic on health system utilization.

Emerging research is exploring the effect of the pandemic on health system utilization. One recent study showed that the pandemic delayed access to pediatric care in Italy, with parental hesitation surrounding viral exposure being a commonly reported deterrent [6]. Similarly, an Austrian observational study noted an unexpected decline in hospital admissions for people with acute coronary syndrome during the COVID-19 pandemic [7]. The authors of that study posited that the observed decrease was influenced by several factors, including fear of infection and strict stay-at-home orders. The reduced use of medical services during a public health crisis is not a new phenomenon. The impact of COVID-19 on medical admissions and research recruitment parallels that of the experience during the 2003 severe acute respiratory syndrome (SARS) outbreak. A Taiwanese study reported large reductions in inpatient care expenditures at the height of the 2003 SARS outbreak, followed by a return to usual levels toward the end of the pandemic, ultimately suggesting that the fear of disease influenced the degree to which people sought care [8]. However, despite these studies, there are insufficient data regarding how the COVID-19 pandemic is specifically impacting the rates of TBI-presenting patients in the ED in the United States.

It is important to understand the effects of the pandemic on ED utilization because pediatric TBI research is a tremendously understudied field. In the parent study, pediatric TBI patients were recruited into a biobank. The biomarkers of these participants were analyzed following blood sample collection. Recruitment of patients occurred within the ED and the research team was unsure how the pandemic would affect the presentation of eligible cases. Furthermore, the COVID-19 pandemic may impact the occurrence of TBI in ways that are not yet understood. For example, the cancellation of school sporting events could lead to a reduction in TBI occurrence (eg, cancellation of contact sports); however, children staying at home due to school closures could lead to an increase in TBI occurrence (eg, rough-housing). There remains a gap in the current literature studying the aforementioned issue surrounding ED utilization in the context of TBI occurrence patterns. To address this gap, the purpose of this study was two-fold. The primary objective was to evaluate changes in ED utilization operationalized by (1) occurrence, (2) location of injury, (3) mechanism of injury (MOI), and (4) age of TBI-presenting patients through relevant electronic health records (EHRs) screened during the pandemic (2020) and the year prior (2019). The secondary objective was to evaluate the efficacy of the research team’s internal screening processes amidst the pandemic.

## Methods

### Ethics Committee Approval

Approval was obtained from The University of Texas at Austin Institutional Review Board (protocol number 2018-04-0018).

### Recruitment

**Figure 1:**
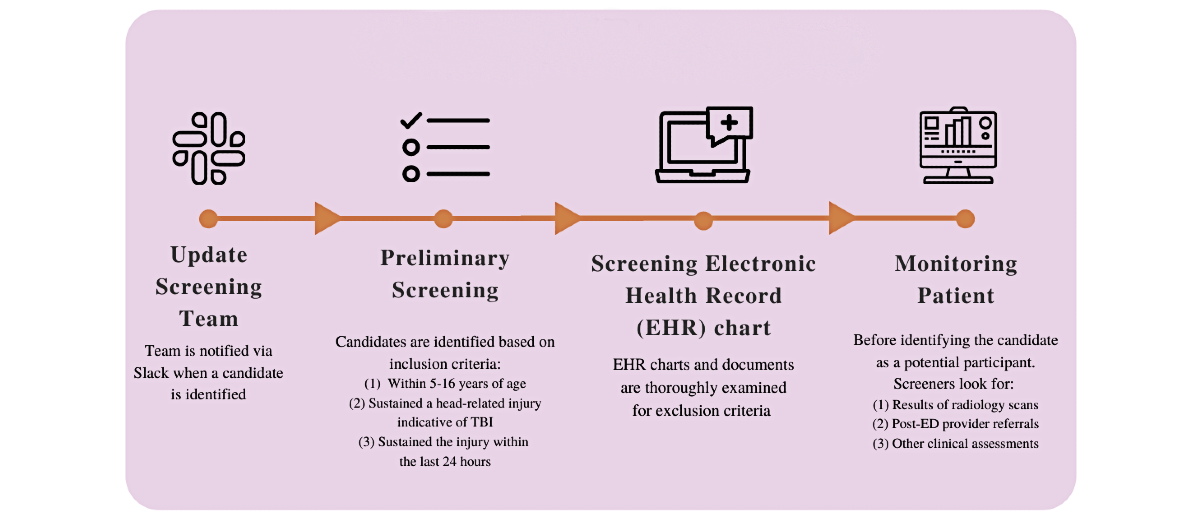
Schematic outlining the 4-part screening process: (1) communication between screener and consenter via Slack, (2) preliminary screening of emergency department (ED) census, (3) screening the EHR, and (4) continued monitoring for relevant updates (eg, radiology scans, referrals). TBI: traumatic brain injury.

This study is a secondary analysis from a larger parent study; the detailed recruitment process is described in Section 2.1 in a previous publication [9] and is resummarized schematically in Figure 1. The parent study, which actively recruited participants from July 2018 to March 2020, focuses on advancing the pediatric TBI knowledge base by analyzing biomarkers in the blood samples of individuals 5-16 years of age who have sustained a TBI and uses pediatric orthopedic injuries as controls.

Enrollment entails a two-step process: screening and consenting. Screeners and consenters obtain Health Insurance Portability and Accountability Act (HIPAA) and Collaborative Institutional Training Initiative certifications prior to accessing patient records. The visit reasons and individual patient charts from the ED census and individual EHRs were used to screen 2321 candidates between March 2019 and August 2020. Relevant data collected from the months of March, April, and July were compared across the 2 years. Screening inclusion criteria included (1) aged 5-16 years, (2) sustained a head injury, and (3) the injury occurred within the last 24 hours. If a candidate did not meet these criteria, they were excluded from any subsequent steps of the recruitment process, as outlined in detail in the previous publication [9]. The charts of candidates deemed potentially eligible were opened in compliance with the HIPAA. A form is completed for screened individuals that reflects when and why the chart was opened. These form responses are aggregated and made available for data abstraction as needed.

Once a candidate is identified, the screener updates any other on-call screeners of the newly identified candidate via Slack, a channel-based messaging platform. Communication via Slack allows for any uncertainties to be resolved, prevents miscommunication with other screeners on-call, and avoids duplicate responses. Under normal circumstances, the consenter on-call will also be notified of a candidate via a screener’s Slack message. The screener and consenter may continue a private-messaging conversation to exchange any further necessary communication in a HIPAA-compliant manner. Subsequently, under normal circumstances, the consenter begins to collect data for the parent study. This consists of three time points: (1) baseline, the day participants were consented and enrolled into the study; (2) 3 months postinjury and enrollment; and (3) 6 months postinjury and enrollment. At each time point, the participant and their parent or guardian answered questions from the same five Patient-Reported Outcomes Measurement Information System (PROMIS) scales and only the participants additionally answered questions from three Quality of Life in Neurological Disorders (Neuro-QoL) scales. In addition, the participant provided a blood sample once at baseline and a saliva sample once at 6 months. However, the COVID-19 pandemic required an adapted recruitment strategy, namely the continuation of screening and the halting of consenting practices and participant enrollment.

### COVID-19 Implications

COVID-19 was designated a global pandemic by the World Health Organization (WHO) on March 11, 2020. The recruitment site affiliated with the parent study halted in-person research activities that did not provide a direct benefit to participants on March 13, 2020. In addition, the National Institutes of Health recommended limiting research-related study visits and nonessential travel [10]. These guidelines contributed to the research team’s decision to halt the consenting process to (1) reduce overall exposure and (2) allow pediatric health care teams to focus on emergent issues without extraneous research activities taking place. Screening, an inherently remote process via the online EHR system, continued with slight modifications. Under normal circumstances, the clinical research team would optimize screener-consenter shift coverage.

Although the team was not consenting candidates, screening served as an accessible avenue for the research study to progress amidst the pandemic and to evaluate the efficacy of newly implemented screening protocols and training. An updated screening training module was created to clarify the scope of the study by refining the inclusion criteria. One such clarification was that if a candidate meets the inclusion criteria but is out of the age range, the screening Google Form should be recorded but the EHR chart should remain unopened. A question was added to the form to identify whether or not a candidate was screened during the COVID-19 pandemic.

### Operationalization and Processing of Variables

Nine variables were examined to determine the effects of the pandemic on internal operations and hospital utilization patterns. The variables used to examine effects on hospital utilization patterns for potential participants were: (1) type of injury, (2) location of injury, (3) MOI, and (4) age. The variables used to examine effects on internal operations were: (1) screened charts, (2) screening coverage, (3) identified candidates, (4) identified potential participants, and (5) identification time. Next, records that did not indicate a potential participant were discarded for the scope of the analyses. The ED visit reasons for the remaining records were broken down into two major categories: (1) “TBI,” representing records that directly indicated a TBI (eg, “TBI,” “Closed Head Injury [CHI],” “Head Trauma”); and (2) “TBI-related orthopedic injuries,” representing records that indicated orthopedic injuries that *may* have been related to a TBI (eg, “Jaw Injury,” “Forehead Contusion”). From these two major categories, the mechanisms of injury were narrowed down to six of the most commonly reported categories: (1) “Fall,” (2) “MVA,” (3) “Strike,” (4) “Assault,” (5) “Unknown,” and (6) “Other.”

The mechanisms of injury written by screeners also included a description of the injury, from which information about the location could be extracted. This was a secondary variable that was created and split into three categories: (1) “Outdoor Injuries,” (2) “Indoor Injuries,” and (3) “Unknown.” The categories were determined by certain key words included in the description. “Inside” injuries for 2019 were classified as being inside at any location (eg, school, office space, private residence). “Outside” injuries for 2019 were classified as any injury that did not occur inside a building (eg, trampoline fall in backyard, MVA). For example, a description of “CHI on playground while running” indicated an outdoor injury, as playgrounds are located outside, and a description of “Slipped and hit head on bed” would indicate an indoor injury, as beds are located inside a building. The breakdown of each variable and their operational definitions are provided in Table 1 and Table 2.

**Table 1:** Screening variables.

Variable^a^	Operationalization	Formula
Screened charts	The total number of screened charts during a given time period	N/A^b^
Screening coverage	The number of hours spent screening per month as a proportion of the total number of hours in a given month^c^; the higher the percentage, the better the screening coverage	(Total number of hours spent screening/total number of hours in a given month)×100
Identified candidates	A person who presented to the ED^d^ whose census data suggest a potential traumatic brain injury and justify opening a screened chart under Health Insurance Portability and Accountability Act guidelines	Proportion of candidates=total number of opened charts/total number of screened charts
Identified potential participants	An individual who, following further chart review, continues to meet the inclusion criteria and would be approached by a consenter	Proportion of potential participants=total number of potential participants/total number of opened charts
Identification time	Length of stay in the ED of a potential participant recorded by the screener at the time of identification, which can reflect how quickly a screener can identify a potential participant; a shorter length reflects a quicker identification time	N/A

^a^Screening variables are defined as variables that provide information about screening patterns from data collected from the emergency department census and individual electronic health records.

^b^N/A: not applicable.

^c^March and July have 31 days, whereas April has 30 days; thus, the total proportion of hours screened was calculated using 744 and 72 total monthly hours for these months, respectively.

^d^ED: emergency department.

**Table 2:** Hospital utilization variables.

Variable^a^	Operationalization	Notes
Age	The average age of potential participants during a given time period	Age was treated as a continuous variable since average age was calculated between 2019 and 2020; therefore, no groupings were used
Type of injury	ED^b^ visit reasons classified into the following two subcategories: (1) indication of possible TBI^c^ and (2) indication of orthopedic injury possibly relating to a TBI	(1) examples include closed head, injury, syncope, and headache; (2) examples include jaw injury, forehead or facial contusion, and cervical spine injury
Mechanism of injury	Collected from the description of the injury and classified into the following five subcategories: (1) fall, (2) motor vehicle accident (MVA), (3) strike, (4) assault, and (5) unknown	A mechanism of injury classified as “unknown” is defined as occurring in an unspecified manner due to lack of details in the EHR^d^
Location of injury	Collected from the description of the injury and classified into the following three subcategories: (1) inside, (2) outside, and (3) unknown	(1) defined as occurring inside at any location (eg, office space, school, private residence); (2) defined as occurring at any defined location other than those defined above as “inside,” such as office space, school, private residence (eg, MVA, riding a bike); (3) defined as occurring in an unspecified location due to the lack of details in the EHR (eg, punch to the head, hit wall, fall from syncope, hit in the back of head by elbow)

^a^Hospital utilization variables are defined as variables that provide information about hospital utilization patterns with data collected only from the potential participants’ electronic health record.

^b^ED: emergency department.

^c^TBI: traumatic brain injury.

^d^EHR: electronic health record.

### Analysis

Screening data were deidentified according to HIPAA guidelines before being recorded and collected through Google Forms, which compiled each screened individual’s collected information into a single “record.” These records were displayed as rows on the imported Google Sheets corresponding to the responses collected through the Google Form. Information not pertaining to data analyses, such as “Screener Name” or “Glasgow Coma Scale Score,” were omitted. Sex was omitted in the analysis because previous screening forms did not collect the participant’s sex before all inclusion criteria were met. Thus, if inclusion criteria were not met, sex, ethnicity, and race were not recorded in the screening form, resulting in incomplete records, which were not used. Screening form responses were downloaded from Google Forms and imported into Google Sheets. The values were then standardized and cleaned of erroneous values, missing data, and duplicate form responses through data-matching methods. Form response time stamps were compared to the potential participant’s time of arrival, MOI, and age to ensure duplicates were removed and missing data were then completed. The Control+F function served to find responses that had similar characteristics so that the data could be manually reviewed one final time. Thus, the records used for analysis contained no missing data and analyses were performed using complete data sets. The sample size for the analyses was determined after (1) discarding all incomplete records and (2) including only those records that indicated potential participants and candidates, as needed for the analysis. For the purposes of this study, a *candidate* is defined as a person who presented to the ED whose census data suggest a potential TBI and justify opening a screened chart under HIPAA guidelines. A *potential participant* is defined as an individual who, following further chart review, continues to meet inclusion criteria and would be approached by a consenter.

Google Sheets was also used to tabulate the frequency of the following variables pertaining only to potential participants: (1) type of injury, (2) MOI, and (3) location of injury. Since the WHO declared COVID-19 a pandemic in March of 2020, data analysis was restricted to the 6-month period between March and August of 2020; analogous data from 2019 were used to compare cumulative responses. The second comparison was performed between specific months corresponding to state-wide mandates from 2020 and 2019, as shown in Table 3. This comparison was performed for the specific months of March, April, and July between 2020 and 2019, as these months corresponded to state-wide COVID-19 mandates, as referenced in Table 1. An *α* value of .05 was used as the a priori cutoff for statistical significance.

All comparisons were assessed in the R programming language [11], using the two-sample Student *t* test and the Mann-Whitney-Wilcoxon rank-sum test to determine any significant differences in the following variables between the years 2019 and 2020: (1) screened charts, (2) identification time, and (3) average age of potential participants. Screening coverage was calculated, by hand, using Google Calendar to track and record screening hours. A two-proportion *z* test was used to compare the hours spent screening and the rates of candidates and potential participants between 2019 and 2020. For potential participants whose visit reason was indicative of a TBI, a two-proportion *z* test was also used to compare the frequencies of MOI, types of injury, and location of injury between 2019 and 2020. The Shapiro-Wilk test was used to determine normality of the two samples and to select the appropriate statistical test (ie, Mann-Whitney-Wilcoxon rank-sum test for samples not normally distributed and Student *t* test for normally distributed samples). Further, an F-test was performed to confirm equal variance of normally distributed samples. Unequal variances were addressed by performing the Welch *t* test. Google Sheets and Canva were used to create data visualizations.

**Table 3:** Timeline of mandates issued by the state of Texas regarding COVID-19 [12].

Date	Protocol implemented
March 24, 2020	The Stay at Home or Place of Residence order became effective as of 11:59 PM. The first executive order was signed, which banned gatherings of 10+ people, closed dine-in restaurants and schools, and limited visitations to long-term care centers.
April 17, 2020	Texas Governor Greg Abbott issued an executive order that in part calls for schools to remain closed for the remainder of the academic year [13].
May 1, 2020	The reopening process began with 25% capacity at most stores and restaurants.
June 26, 2020	Lockdown orders were reimplemented, with capacity being dropped to 50% at most locations and bars being shut down. Six days later, a mask mandate was instituted.

## Results

In total, 1245 screening form entries from 2019 and 1076 screening form entries from 2020 were analyzed. Table 4 provides comparisons of different screening variables between analogous months. The proportion of candidates in 2019 was significantly greater than that in 2020 (*P*<.001). Specifically, the proportion of candidates in April 2019 was significantly greater than that in April 2020 (*P*=.01). The proportion of potential participants in 2019 was significantly lower than that in 2020 (*P*<.001). The proportions of potential participants across all measured time points in April, March, and July were significantly lower in 2019 than in 2020, as seen further in Table 4. The average age of potential participants was significantly older in 2019 by 1.62 years (*P*=.006).

**Table 4:** Screening and hospital utilization patterns.^a^

Time period			Proportion of screening hours^b^		Total screened charts, n		Total opened charts, n		Proportion of candidates, %		Proportion of potential participants, %		Total length of stay (minutes)		Mean age of potential participants (years)
**All months (March, April, and July)**															
	2019	2392		1245		908		72.9		21.4		37		8.93	
	2020	2783.8		1076		633		58.8		35.1		35		7.31	
	*P* value	.09		.38		.05		<.001		<.001		.48		.006	
**March**															
	2019	46.6		284		218		76.8		23.9		32		10	
	2020	32.5		66		46		69.7		43.5		55		10	
	*P* value	<.001		.07		.08		.30		.006		.03		>.99	
**April**															
	2019	65.3		214		157		73.4		17.2		36		10.22	
	2020	84.9		188		117		62.2		29.9		33		9.63	
	*P* value	<.001		.09		<.001		.01		.01		.77		.43	
**July**															
	2019	54.3		134		91		67.9		26.4		42		8.23	
	2020	69		209		125		59.8		39.2		22		9.43	
	*P* value	<.001		.22		.42		.16		.07		.01		.37	

^a^See Table 1 and Table 2 for definitions and formulas for each variable.

^b^Presented as total raw numbers screened for the respective analyzed months for the All Months category and as percentages (raw number/total number of hours) for the individual month categories.

**Figure 2:**
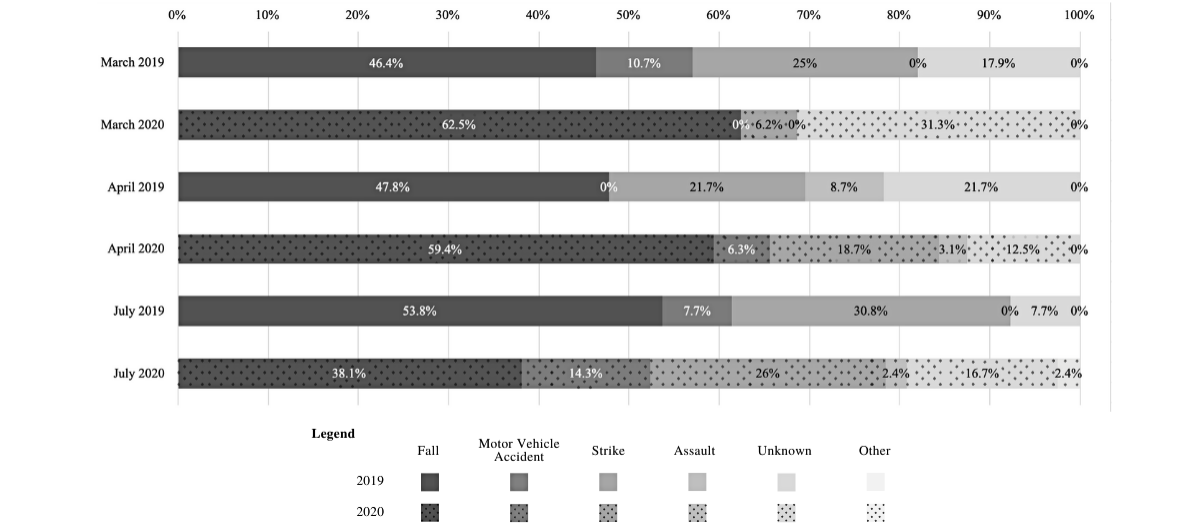
Mechanism of injury classifications of potential participants in March, April, and July of 2019 vs 2020.

Table 5 displays a comparison of the different types of injuries that were reported for potential participants through the ED census. There was a significantly greater number of potential participants who had visit reasons explicitly indicating a TBI (*P*<.001) and those with an orthopedic injury possibly related to a TBI (*P*=.002) in 2020 than for those who came into the ED for the same reasons in 2019, respectively. Table 5 also displays the general location that potential participants were injured in (ie, outside, inside). There were significantly more injuries that occurred inside in 2020 than those in 2019 (*P*=.02). When examining the various MOI, no significant differences were found between 2019 and 2020. There was a higher frequency of visit reasons indicating an MOI of the Fall and Strike categories in 2020 than in 2019; however, these differences were not statistically significant. A detailed comparison of the MOI during the months of interest across the 2 years can be found in Figure 2.

One key finding was that approximately 55 minutes passed before a screener identified a candidate in March 2020, which was significantly slower than the 32 minutes that passed in March 2019 (*P*=.03). Approximately 22 minutes passed before a screener identified a candidate in July 2020, which was significantly faster than the 42 minutes that passed in July 2019 (*P*=.01). A total of 241.8 hours were utilized for screening in March 2020, which was significantly less than the 346.5 hours utilized for screening in March 2019 (*P*<.001). In April 2020, 611.5 hours were utilized for screening and 513.5 hours were utilized in July 2020. Both of these monthly totals were significantly greater than the total hours utilized for screening in the analogous months in 2019: 470.5 hours in April (*P*<.001) and 404.3 hours in July (*P*<.001).

**Table 5:** Emergency department (ED) visit reason, mechanism of injury, and location of injury of potential participants.

Characteristic		2019^a^ frequency, n (%)	2020^a^ frequency, n (%)	*P* value
**ED visit reason**				
	Total participants^b^, n	195	222	N/A^c^
	TBI^d^	103	181	<.001
	TBI-related orthopedic injury^e^	3	18	.002
**Mechanism of injury**				
	Total participants, n	103	181	N/A
	Fall	47 (45.6)	104 (57.5)	.07
	MVA^f^	4 (3.9)	13 (7.2)	.39
	Strike	32 (31.1)	37 (20.4)	.06
	Assault	3 (2.9)	3 (1.7)	.78
	Unknown	17 (16.5)	23 (12.7)	—^g^
	Other	0 (0)	1 (0.5)	—
**Location of Injury**				
	Total participants, n	103	181	N/A
	Inside injuries	11 (10.7)	39 (21.6)	.02
	Outside injuries	49 (47.6)	77 (42.5)	.38
	Unknown location	43 (41.7)	65 (35.9)	—

^a^The years of 2019 and 2020 in this subanalysis are defined to be the months of March, April, and July of each year, respectively.

^b^Indicates the total number of flagged potential participants, including those in the non-TBI-related orthopedic injury and unknown categories, which were not included for comparison.

^c^N/A: not applicable.

^d^TBI: traumatic brain injury.

^e^These visit reasons include orthopedic injuries that may be related to a TBI (eg, jaw injury, forehead contusion).

^f^MVA: motor vehicle accident.

^g^Statistical analysis was not possible for unknown groups since this was an indefinite category.

## Discussion

### Principal Findings

Regarding ED utilization patterns, significantly older patients were flagged as potential participants in 2020 than in 2019. Significantly more potential participants came into the ED with TBI or TBI-related injuries in 2020 than in 2019, and the MOIs for these potential participants were not significantly different between the two years. There were varying outcomes among the analyzed months regarding evaluation of the efficacy of the internal screening process. Generally, positive effects of the change in internal screening protocols were evident, as measured by analyzed variables such as total screened charts, total opened charts, length of stay (LOS), and total hours spent screening. These findings are discussed in detail further.

The novelty of this study lies in the quick adaptation of the research team’s previously established ED tracking protocol to identify potential participants, allowing for examination of the novel effects of the pandemic on ED utilization. Although the research team was no longer consenting and enrolling participants, the internal screening processes proved to be beneficial during the pandemic as the screening could occur concomitantly amidst state-regulated mandates (eg, stay-at-home orders, social distancing recommendations). Although many research studies halted procedures during the pandemic, the preexisting structure of the parent study allowed the research team to examine ED utilization patterns and evaluate the adaptability of the established screening processes with minimal changes to the existing modality.

### ED Utilization Patterns

The average age of a potential participant was significantly older in 2020 (8.93 years) than in 2019 (7.31 years) by approximately 1.5 years (*P*=.006). Notably, in 2019 and 2020, the average age fell in the middle childhood range (6-8 years) as defined by the Centers for Disease Control and Prevention (CDC) [14]. One study noted that children who suffered a TBI during this critical developmental stage were vulnerable to poorer cognitive outcomes as compared to children in other developmental stages [15]. This finding could be related to the level of health care avoidance that is seen in parents during the pandemic, as several studies have supported this hypothesis [16,17]. Although further details about the profiles of such parents are yet to be studied in the United States, it can be hypothesized that parents may be more hesitant to bring a younger child into the ED, which might be perceived as a high-risk environment.

Significantly more potential participants came into the ED with either a TBI (*P*<.001) or TBI-related orthopedic injuries (*P*=.002) in 2020 than in 2019, as seen in Table 5. In response to an executive order issued by Texas Governor Greg Abbott calling for all schools to remain closed for the remainder of the 2020 academic year, all nine independent school districts in the Austin area transitioned to online learning, forcing many students to conduct their education at home [13]. Child sports play activity also decreased after lockdown measures, leading to increased home-based activities [18]. It is hypothesized that quarantine measures may have increased TBI or TBI-related orthopedic injuries that occurred at home. This hypothesis is supported by the significant increase in injuries that occurred inside in 2020 when compared with those in 2019 (*P*=.02).

The proportion of the various MOIs seen in Table 5 from potential participants who came in for a TBI did not significantly differ between years. The lack of significance in the proportion of MVAs reported as the MOI in 2020 when compared to 2019 is interesting, as there were fewer MVAs reported in Austin according to one study [19] and notably less traffic occurring in 2020 overall. There was 40% less traffic congestion recorded in response to the Declaration of State Disaster issued on March 13, 2020, as an effort to “mitigate the spread of COVID-19,” when compared to the same day in 2019 [20]. The more severe order of restriction, Stay at Home or Place of Residence, became effective March 24, 2020, resulting in 70% less traffic per day on average, which continued to be true throughout April (76.46%) and July (71.84%), following the significant events described in Table 3*.*

### Evaluating the Efficacy of Internal Screening Processes

The lack of significance for total screened charts recorded by screeners across 2019 and 2020 may be explained in part by the fact that the recruitment site is not a COVID-19 treatment facility, possibly limiting the impact of the pandemic on ED admissions relating to the study’s inclusion criteria. However, given the potentially serious nature of a head injury, any concerns about contracting the COVID-19 virus might have been disregarded. The utilization of COVID-19 treatment facilities as compared to non-COVID-19 treatment facilities remains to be empirically studied.

However, of the total screened charts, there were significantly less charts opened in April 2020 (117 charts) when compared to those opened in April 2019 (157 charts), which may be related to screening protocol changes (*P*<.001). Examination of previous data showed common visit reasons that screeners would pursue (eg, facial lacerations), but ultimately did not result in TBI once the chart was further examined. Based on reports from the CDC, screening training was updated to ensure that screeners would only examine charts with injuries more likely to be indicative of a TBI (eg, fall). According to the CDC, the leading causes of TBI include falls, strikes, MVA, and intentional self-harm [21].

The significant increase in the LOS screening variable during March 2020 (55 minutes) compared with that in March 2019 (32 minutes) could be explained by the difference in screening coverage between the two analogous months (*P*=.03). If there is consistent coverage throughout the day, there is a higher probability of a candidate being identified quickly and thus a shorter LOS reported by the screener. Since a large majority of the research volunteers are undergraduate students, screening coverage tends to decrease during spring break; however, the impact was greater during 2020 due to the extension of the spring break, as mandated by the university in an effort to adapt to the pandemic. The proportion of total hours spent screening during March 2020 (346.5 hours) was significantly less than the proportion of total hours spent screening during March 2019 (241.8 hours; *P*<.001). This discrepancy could be attributed to the time it took for the research team to modify its recruitment protocols to adapt to the ongoing changes to safety regulations. The research team does not require its screeners to continue screening throughout academic breaks, thus contributing to the decrease in screening coverage seen in March 2020.

After the recruitment site and the university halted human-subjects research on March 13, 2020, and March 15, 2020, respectively, the research team expeditiously decided to convert all consenting shifts into screening shifts. This decision positively impacted screening coverage in April 2020. There was a significant increase in the proportion of hours spent screening in April 2020 (611.5 hours) when compared to April 2019 (470.5 hours; *P*<.001). Additionally, there was no significant difference in the LOS between April 2020 (33 minutes) and April 2019 (36 minutes; *P*=.77), demonstrating the research team’s efficient transition of recruitment practices.

There was a significant increase in the total number of hours spent screening in July 2020 (513.5 hours) when compared to July 2019 (404.3 hours). The research team recruited a new cohort of research assistants in May 2020, all of whom were trained to screen quickly and efficiently. With enrollment efforts still halted, the research team encouraged the new cohort to begin screening in the summer, thus increasing screening coverage during a time that otherwise experiences a decrease in coverage. This also positively impacted the LOS during July, as there was a significant decrease in LOS in July 2020 (22 minutes) compared to July 2019 (42 minutes; *P*=.01).

In addition to improved screening coverage, there was greater accuracy with respect to more charts being opened for further screening where the individual was deemed eligible to approach. Although there was a greater proportion of candidates (charts opened) in 2019 than in 2020 (*P*<.001), there was a greater proportion of potential participants (cases deemed approachable) in 2020 than in 2019 (*P*<.001). The proportion of potential participants was significantly greater overall in 2020 than in 2019 (*P<*.001) and at every measured time point. This further reflects the need for continued enrollment efforts and highlights the impact of the pandemic on the biobanking efforts of the parent study. The improvement in screening accuracy may be due to changes in training and laboratory protocols; however, this remains to be empirically studied.

### Strengths and Limitations

Due to the unprecedented nature of the pandemic, the study was executed using the parent study’s previously established protocols. Consequently, one study limitation is the inability to sufficiently evaluate the effects of the pandemic, as the data collection methods were originally not tailored to the scope of this study’s research questions. The research team could have adapted the methods of data collection to better identify the effects of the pandemic. Collecting data on the length of time that elapsed between injury and the ED visit, whether or not parents were aware that the hospital was not treating COVID-19 patients, and whether or not parents felt comfortable bringing their children in are a few questions that may elucidate how parental behavior may have been impacted by the pandemic. Additionally, collecting data on demographic variables (eg, race, ethnicity) of candidates who visit the ED can help to explore the relationship between hospital utilization rates during a pandemic and other social factors. This is an important trend to study as reports of health disparities and inequities during the pandemic across specific ethnic and race groups become more evident [22].

Although efforts were made to clarify the scope of the study and to refine the inclusion criteria, the study is limited by the precision of the screeners monitoring the EHR and decisions made to open a potential participant’s chart. Imprecision may lead to a greater number of potential participants to be included that did not actually meet the inclusion criteria or fewer potential participants, depending on whether a particular screener was more lenient or stringent when determining potential participants. This could have led to inflation or underestimation of the reported values. Additionally, intraobserver bias is possible due to differences in screener experience of each research assistant.

### Conclusions and Future Directions

Findings from this study indicate significantly higher ED visits for a TBI during the COVID-19 pandemic during 2020 when compared to the analogous months of 2019 (*P<*.001). Based on these findings, it is plausible for the parent study’s enrollment efforts to continue, albeit with added precautions, including: (1) socially distant consenting protocols or virtual consenting, (2) smaller teams of consenters with alternating shifts, (3) precautions such as daily symptom tracking and biweekly COVID-19 tests required for on-site consenters, and (4) requiring on-site consenters to receive the COVID-19 vaccine before returning to their roles. This information is relevant to the research community from a number of perspectives, including funding, tenure, publications, and deliverables during a pandemic. If these precautions cannot be met, the research team must consider remote data collection from clinical studies to continue a steady workflow amidst global change.

As the research team continues to systematically gather data for the parent study, future directions can focus on developing dynamic protocols to reflect relevant public health issues as they evolve, including: (1) modifying data collection protocols, (2) employing effective methods of tracking health care system utilization, (3) understanding the factors that influence a participant’s decision to enroll, and (4) understanding the factors that affect participant retention. For existing records collected prior to this analysis, former participants might be contacted to request additional information, thus filling in missing information. Another avenue to reduce risk of infection among in-person consenters might be to postpone sample collection (eg, saliva) until state regulations suggest specimens can be collected safely. This study illuminates the importance of continuous data collection, allowing the research team to adapt existing protocols to combat unexpected changes such as the COVID-19 pandemic.

## Abbreviations

CDC: Centers for Disease Control and Prevention
CHI: closed head injury
ED: emergency department
EHR: electronic health record
HIPAA: Health Insurance Portability and Accountability Act
LOS: length of stay
MOI: mechanism of injury
MVA: motor vehicle accident
Neuro-QoL: Quality of Life in Neurological Disorders
PROMIS: Patient-Reported Outcomes Measurement Information System
SARS: severe acute respiratory syndrome
TBI: traumatic brain injury
WHO: World Health Organization
